# Intention and practice on personal preventive measures against COVID-19 among older adults in the Kingdom of Saudi Arabia: an epidemiological study using the Theory of Planned Behaviour

**DOI:** 10.3389/fpubh.2023.1315443

**Published:** 2023-12-14

**Authors:** Reem S. AlOmar, Amal S. AlHarbi, Layla A. Abu Abdullah, Sarah M. Almuqbil, Zahra S. Albahrani, Hawra M. Aldar, Fatimah S. Alzouri, Manar A. Al-Shiban, Nouf A. AlShamlan, Marwa M. Shafey, Assim M. AlAbdulKader, Nijr S. Alotaibi

**Affiliations:** ^1^Department of Family and Community Medicine, College of Medicine, Imam Abdulrahman Bin Faisal University, Dammam, Saudi Arabia; ^2^College of Medicine, Imam Abdulrahman Bin Faisal University, Dammam, Saudi Arabia; ^3^National Program for Community Development – Tanmiah, Riyadh, Saudi Arabia

**Keywords:** public health, epidemiology, Theory of Planned Behaviour, older adults, COVID-19

## Abstract

**Introduction:**

Older adults aged 65 years and above are among the most vulnerable to adverse outcomes and death following a COVID-19 infection. The weekly epidemiological updates by the World Health Organisation show that the continued emergence of concerning subtypes of the virus indicates that the pandemic remains a public health concern and the public should continue to comply with personal preventive measures (PPMs). This study applies the Theory of Planned Behaviour (TPB) which is rooted in the field of Public Health, Epidemiology, and Preventive Medicine to Saudi older adults to predict their health behaviour.

**Methods:**

This behavioural epidemiological study recruited older adult participants aged 65 years of age and above. A tool which consisted of sociodemographic and health-related questions, as well as questions regarding the components of the TPB, namely, Attitude, Subjective Norm, Perceived Behavioural Control was used. Bivariate analyses, followed by unadjusted and adjusted multivariable logistic regression analyses were performed to derive odds ratios and 95% confidence intervals.

**Results:**

The total number of participants was 502. The mean age was 70.34 years, with similar distributions between males and females. In total, 52.2% intended to practice PPMs, whereas only 48% had a good practice. Also, 56% had a favourable Attitude towards PPMs, 61.4% had a positive Subjective Norm and 39.8% had perceived they had a high control over their behaviour. Females, and high educational status were predictors for high intention to practice PPMs (OR = 1.59, 95% CI = 1.01–2.52 and OR = 2.72, 95% CI = 1.44–5.16 respectively). Further predictors included Attitudes, Subjective Norm and Perceived Behavioural Control. Results also show that intention to practice was significantly associated with a lower odd of practicing PPMs (OR = 0.06, 95% CI = 0.04–0.10).

**Conclusion:**

Current findings highlight the need to continue with public health efforts targeting vulnerable older adults. Also, the fact that intention negatively predicted practice highlights the need for further behavioural epidemiological studies addressing the intention-behaviour gap.

## Introduction

Disease prevention is crucial in various aspects of life and health. Ever since the emergence of the coronavirus disease – 2019 (COVID-19) in late December of 2019, and it being announced as a pandemic in March of 2020, over 770 million confirmed cases and almost 7 million deaths have been registered (1, 2). This disease originates from a single-stranded RNA virus that is able to cause respiratory, gastrointestinal and central nervous system infections in its host (3). Due to the virus’s ability to rapidly spread and evolve, it remains a public health priority to this day.

Very early on, specific groups of people were understood to be at a higher risk of mortality after a COVID-19 infection. These groups include older adults aged 65 years of age and above, patients with comorbidities as well as immunocompromised patients (4, 5). Therefore, preventive measures have been put in place, with the World Health Organisation (WHO) leading global efforts by setting up periodic bulletins with guidelines, as well as establishing the COVID-19 dashboard, and the United Nations supporting national preparedness and response plans to countries worldwide (1, 6). During the early stages of the pandemic, and while vaccines were still under development, the primary focus was on non-pharmaceutical interventions (7). These were steps that could be taken to mitigate and control the spread of the disease, and consequently alleviate its burden and allow time to develop the much-needed vaccines and treatments.

The Kingdom of Saudi Arabia (KSA) was among the first countries to proactively implement preventive measures that were – in those early stages – considered unprecedented. For example, two months before the detection of any cases in the country, a national committee with members from different governmental agencies had been entrusted with overviewing global updates and providing recommendations in preparing for the possibility of cases locally (8). Upon the discovery of the first case, all entry points to the two holy cities of Makkah and Madinah were suspended and international flights were cancelled, shifting schools and other educational institutions to remote learning as well as nationwide curfews (8, 9). Furthermore, health campaigns over official governmental social media accounts and text messages were streamed daily to raise public awareness and to remind everyone to abide by national preventative measures (8, 10). These measures have greatly assisted in reducing and controlling the spread and potential further burden of the disease (9).

The Theory of Planned Behaviour (TPB) is a theory that attempts to draw the framework for predicting adherence and compliance to preventive measures (11). This theory is a conceptual model that was established in 1980 as an extension of the Theories of Reasoned Action to explain the effect of information and motivation on behaviours (12).

Even though this theory has been extensively studied in several countries, very few studies within the KSA are found, and none have studied older adults specifically and/or preventive measures in general. For example, it has been applied to dental healthcare workers to examine the factors associated with infection control behaviour (13), as well as among the general public to study their intent to receive the COVID-19 vaccine alone (13, 14). Although on the 5th of March of 2022, all restrictions have been scraped, the continued emergence of concerning subtypes of COVID-19 and the fact that older adults remain at risk of complicated outcomes and death compounded by the lack of research on this specific vulnerable population has given rise to this research.

Therefore, this study will first provide a theoretical foundation for the TPB within the context of COVID-19, and subsequently apply the components of the theory to Saudi older adults to examine their intention to practice personal preventive measures (PPMs) as set out by the Saudi health authority guidelines. The study will also discuss the study’s findings and provide implications for public health policy.

## Theoretical foundation

Several theories that attempt to predict health behaviours are available in the medical literature. These include – but are not limited to – the Health Belief Model, Technology Acceptance Model, and the TPB (11, 15, 16). The TPB argues that behaviour is driven by the intention to perform that particular behaviour forming what is known as the individual’s “belief structure” (11). In the case of COVID-19 preventive measures, this structure would be comprised of Attitude towards preventive measures (i.e., their perceived necessity), Subjective Norms (i.e., whether others support and perform these measures), and Perceived Behavioural Control (i.e., the extent of which the preventive measures are within the individual’s control).

The TPB is related to the science of behavioural epidemiology which has been emerging since the late 1970s (17, 18). Behavioural epidemiology is under the umbrella of Public Health and consists mainly of two concepts, the first is to identify the epidemiological relationship between individual behaviour and disease occurrence, and the second is the epidemiological study of the actual behaviour itself (19). There is a vast literature supporting the application of this theory to predict health behaviour in general and for COVID-19 specifically (12, 18, 20–22).

## Materials and methods

### Study design, setting, and study participants

This behavioural epidemiological study employed a cross-sectional design and was conducted in the Eastern region of the KSA. The study setting were community health centres where older adult participants attended routine physical examinations. Eligibility for inclusion included both male and female participants who were aged 65 years and above, and that they were clear from any neurocognitive disorders such Parkinson’s disease and dementia and were capable of communicating verbally.

### Ethical considerations

The Imam Abdulrahman Bin Faisal University’s Institutional Review Board approved the study (IRB-2022-01-294). The participation was voluntary and there was no requirement to obtain personally identifiable information. Consent to participate was obtained from all participants. The study complied with the principles of the Declaration of Helsinki.

### Sample size and sampling technique

The minimum required sample size was 383. This was based on a previous study in which the intention to practice PPMs was 52% (22), with a precision of 5% and at an alpha level of 0.05. The Epi info software version 7.0 was used for sample size calculations. Since the study targets a delicate population, a non-probability sampling technique was used to recruit participants.

### Data collection tool and processes

The questionnaire was adapted from the WHO Survey tool and guidance and incorporated recent international literature that had also used the TPB within the context of COVID-19 (14, 20–24). Since these studies were either on the general population or on specific health workers, the authors had to adapt the tool to better represent the older adults’ population who are the focus of this work. Three experts namely, a geriatrician, preventive medicine consultant and a public health consultant had reviewed the tool to check for its clarity and appropriateness. Then, a pilot study was performed on a sample of 10 participants aged 65 years and above, all questions were clear and the average time to complete the tool was 8 min.

The first set of variables in the tool included questions on sociodemographic and health-related characteristics. Also, the tool asked whether the participants knew of a previous COVID-19 infection, and if yes, the perceived severity level of that infection (mild, moderate or severe, i.e., hospitalised). The second set of variables were pertaining to the TPB. These included questions on the three main elements of the theory, namely, Attitude towards PPMs, Subjective Norm and Perceived Behavioural Control. Attitudes may be defined as the degree to which an individual holds a favourable or unfavourable assessment of a particular behaviour. Subjective Norm is the belief of whether other people within the community approve or disapprove of a particular behaviour, whereas Perceived Behavioural Control is the perceived difficulty or easiness of performing that behaviour (11). PPMs against COVID-19 may be defined as those measures that are known to protect and prevent infection and which included the following six preventive recommendations; handwashing with soap and water for a minimum of 20 s, avoid touching eyes, mouth, and nose when hands are not washed, staying at home if sick, covering mouth and nose when coughing and sneezing, physical distancing and self-isolation. Furthermore, the tool asked the participants on their intention to practice these PPMs and whether they already do so in the present time.

Data were collected through an online tool which allowed the team to share the link within themselves during the data collection period. In order to protect both the older adult participants and the team, guidelines were followed in terms of respiratory etiquette and distancing.

### Measurements

The Attitude to practice PPMs was measured using the six main recommendations stated above on a five-point Likert’s scale. The mean score was computed for each participant, and those who score the overall mean and above were considered as having a good Attitude, whereas those who scored below the mean were considered to have a poor Attitude. As for Subjective Norm, it was based on two questions, namely, if you were infected with COVID-19, would you let people know? and how much do you trust the prevention information issued by the MoH? These were both measured on the five-point Likert’s scale. Similar to Attitude, participants scoring above the mean were considered to have a positive Subjective Norm and participants scoring below the mean were considered to have a negative Subjective Norm. The Perceived Behavioural Control construct was similarly computed and was based on three questions, namely, do you think COVID-19 would have a serious impact on you and your family? Are you still worried about COVID-19 and other large infectious diseases? And do you have the confidence to protect yourself and your family against COVID-19 and other large scale infectious diseases? (22, 24).

Intention to practice PPMs was measured based on the above six PPM questions, and further questions were added that included the use of antibiotics to prevent and treat COVID-19. These were measured on a Likert’s scale and those who were intending had scored above the mean. The current practicing of PPMs was a simple yes/no response. This allowed us to differentiate between the intention and actual practicing of these measures.

### Data analyses

Descriptive statistics were computed as frequencies and percentages for categorical variables and means ± standard deviations for continuous variables. The study had two outcomes, the first was intention to practice PPMs and the second was the current practice. Bivariate associations were performed to study the associations between these outcomes and all elements of the TPB as well as sociodemographic and health-related characteristics. Both unadjusted and adjusted binary logistic regression analyses were performed for the two outcomes of the study to compute the Odds Ratios (ORs) and their accompanying 95% confidence intervals (CIs). The level of significance was set at 0.05. All analyses were performed in Stata Statistical Software version 15.0 (Stata Corp) (25).

## Results

### Sociodemographic and health characteristics of study participants

The study included 502 participants. The mean age was 70.34 years ±5.85 years. The distribution of males and females were very similar (49 and 51% respectively). The majority of participants (44.40%) had a high school degree and only 1.60% were postgraduates. Only 2.80% reported that they once were healthcare professionals. With regards to their health status, 75.30% reported the presence of a chronic disease condition. Also, 4.60% were living alone and 45.60 of the total sample reported a “good” financial status, compared to only 2% who had reported a bad financial status.

In total, 87.30% had known people who were previously infected with COVID-19, and 42.8% had known someone who had died from it. With regards to personal history, 47.60% reported a previous COVID-19 infection, of those sub-sample of participants, 15.5% were hospitalised due to the severity of the infection (Table 1).

**Table 1:** Sociodemographic and health characteristics of the older adult participants.

Characteristics	*N* (%)
**Age (μ, α)**	70.34 (5.85)
**Sex**	
Males	246 (49.00)
Females	256 (51.00)
**Level of education**	
Read and write	145 (28.90)
High school	223 (44.40)
University graduate	126 (25.10)
Postgraduate	8 (01.60)
**Health care professional**	
Yes	14 (02.80)
No	488 (97.20)
**Chronic disease conditions**	
Yes	378 (75.30)
No	124 (04.70)
**Living circumstances**	
Alone	23 (04.60)
With a family	479 (95.40)
**Perceived financial status**	
Bad	10 (02.00)
Fair	196 (39.00)
Good	229 (45.60)
Excellent	67 (13.30)
**Know someone previously infected with COVID-19**	
Yes	438 (87.30)
No	64 (12.70)
**Know someone who had died from COVID-19**	
Yes	215 (42.80)
No	287 (57.20)
**History of previous COVID-19 infection**	
Yes	239 (47.60)
No	263 (52.40)
**Severity of previous COVID-19 infection** ^**a**^	
Mild	102 (42.70)
Moderate	100 (41.80)
Severe (Hospitalised)	37 (15.50)
**Test confirmed previous infection** ^**a**^	
Yes	201 (84.10)
No	38 (15.90)

a Frequencies and percentages are based on the 239 participants who had reported a previous COVID-19 infection.

### Subjective Norm and Perceived Behavioural Control and Attitude of the study participants

The mean score for the Subject Norm was 8.44 ± 1.68 (range 2–10). In total, 61.4% had a positive Subjective Norm. As for the Perceived Behavioural Control, the mean was 8.33 ± 2.01 (range 3–13) and 39.8% perceived that they had a higher control in their behaviour. Whereas for the Attitude, the median score was 25.51 ± 4.83 (range 6–30) and 56% were found to have a favourable Attitude (Table 2).

**Table 2:** Attitude towards personal preventive measures against COVID-19 among older adult participants.

Personal preventive measures (PPMs)	Attitude *N* (%)				
	Strongly disagree	Disagree	Not sure	Agree	Strongly agree
Washing hands with soap and water for at least 20 s	15 (03.00)	39 (07.70)	28 (05.60)	150 (29.90)	270 (53.80)
Avoid touching eyes, mouth and nose with unwashed hands	12 (02.40)	35 (07.00)	22 (04.40)	162 (32.20)	271 (54.00)
Staying at home (sick or having cold)	21 (04.10)	40 (08.00)	31 (06.20)	159 (31.70)	251 (50.00)
Covering mouth and nose when coughing or sneezing	8 (01.60)	15 (03.00)	14 (02.80)	151 (30.10)	314 (62.50)
Keep physical distancing	26 (05.20)	71 (14.10)	47 (09.40)	139 (27.70)	219 (43.60)
Self-Isolation	12 (02.40)	27 (05.40)	13 (02.60)	127 (25.30)	323 (64.30)
**Overall Attitude towards PPMs**					
Favourable			281 (56.00)		
Unfavourable			221 (44.00)		

Likewise, along the range of the five degrees of Attitudes towards the PPMs, over half the participants (50.00 to 64.30%) strongly agreed to most of them, except for physical distancing which showed the least amount of agreement (43.60%). Physical distancing also showed a higher proportion of strong disagreement compared to other statements (05.20%).

### Intention and practice on PPMs against COVID-19

The mean score for the intention to practice PPMs was 29.44 ± 8.56 (range 10–50). In total, 52.2% had intended on practicing the stated preventive measures. Along the range of the five degrees of intention, 50.8% had very likely intended to practice washing hands with soap and water for at least 20 s. Participants reported lesser percentages of being very likely intending to avoid touching eyes, mouth, and nose with unwashed hands (44.20%), as well as for using disinfectants to clean hands when soaps are unavailable (35.3%). A large proportion of participants exhibited being very unlikely to intend to use antibiotics to prevent or treat COVID-19 (62.70 and 59.40% respectively) (Table 3).

**Table 3:** Intention and practice of personal preventive measures against COVID-19 among older adult participants.

Personal preventive measures (PPMs)	Intending to practice *N* (%)					Practice *N* (%)	
	Very unlikely	Unlikely	Not sure	Likely	Very likely	Yes	No
Wash hands with soap and water for at least 20 s	19 (03.80)	75 (11.40)	21 (04.10)	150 (29.90)	255 (50.80)	402 (80.10)	100 (19.90)
Avoid touching eyes, mouth and nose with unwashed hands	32 (06.40)	81 (16.10)	15 (03.00)	152 (30.30)	222 (44.20)	357 (71.10)	145 (28.90)
Use disinfectants to clean hands when soaps are unavailable	83 (16.50)	93 (18.50)	18 (03.60)	131 (26.10)	177 (35.30)	284 (56.60)	218 (4340)
Disinfecting touchable surfaces	103 (20.50)	101 (20.10)	26 (05.20)	140 (27.90)	132 (26.30)	333 (46.40)	269 (53.60)
Wearing masks in public places	85 (16.90)	96 (19.10)	18 (03.60)	153 (30.50)	150 (29.90)	284 (56.60)	218 (43.40)
Avoid social gathering	148 (29.50)	159 (31.70)	32 (06.40)	104 (20.70)	59 (11.70)	182 (36.30)	320 (63.70)
Ensure physical distance in public places	80 (15.90)	121 (24.10)	18 (03.60)	141 (28.10)	142 (28.30)	288 (57.40)	214 (42.60)
Staying at home (when sick or with a cold)	179 (35.70)	171 (34.10)	27 (05.40)	79 (15.60)	46 (09.20)	111 (22.10)	391 (77.90)
Use antibiotics to prevent COVID-19	315 (62.70)	128 (25.50)	30 (06.00)	16 (03.20)	13 (02.60)	21 (04.20)	481 (95.80)
Use antibiotics to treat COVID-19	298 (59.40)	123 (24.40)	28 (05.60)	31 (06.20)	22 (04.40)	35 (07.00)	467 (93.00)

With regards to the actual practice of PPMs, the analysis found that the mean practice score was 15.62 ± 2.58 (range 10–20) and the proportion of participants who had good practice were 48.0%. Hand washing with soap and water for at least 20 min was reported among 80.1%, avoid touching eyes, nose, and mouth with unwashed hands was stated among 71.1%. The least commonly reported practice was using antibiotics to prevent COVID-19 (7.0%).

### Factors associated with the intention to practice PPMs against COVID-19 infection

Results shows statistically significant differences in the intention to practice PPMs in relation to age, level of education, Attitude towards PPMs, Subjective Norm, and Perceived Behavioural Control (*p* < 0.05). Given these results, Table 4 shows unadjusted and adjusted logistic regression analyses of participants’ characteristics in relation to intention to practice PPMs. The odds of intention were significantly lower among the ≥70-year age group, although this was not significant in the adjusted model. Sex arose as a factor where females were 59% more likely to exhibit intention to practice PPMs (95% CI = 1.0–2.52). Also, the results show that the odds of intention increased with higher education. With respect to the studied four components of the TPB, it was observed that participants who had a favourable Attitude were 4.41 times more likely intending to practice PPMs (95% CI = 2.89–6.73). Also, higher odds were seen among participants who had a positive Subjective Norm (95% CI = 1.53–3.65). Similarly, the odds of intention were 72.3% times higher among those participants with a high Perceived Behavioural Control compared to their counterparts.

**Table 4:** Unadjusted and adjusted binary logistic regression analyses of older adult participants predictors of the intention to practice personal preventive measures against COVID-19 infection.

Predictors	Intention to practice PPMs		Unadjusted OR	95% CI	Adjusted OR	95% CI
	Not Intending *N* (%) 240 (47.80)	Intending *N* (%) 262 (52.20)				
**Age**						
< 70	120 (42.40)	163 (57.6)	*Ref*			
≥70 years	120 (54.80)	99 (45.20)	0.60	0.42–0.86	0.76	0.49–1.16
**Sex**						
Males	119 (49.58)	126 (51.20)	*Ref*			
Females	121 (50.42)	136 (53.10)	1.07	0.76–1.53	1.59	1.01–2.52
**Level of education**						
Read and write	85 (58.60)	60 (41.40)	*Ref*			
High school graduate	104 (46.60)	119 (53.40)	1.62	1.06–2.47	2.17	1.28–3.70
University/Postgraduate	51 (38.10)	83 (61.90)	2.36	1.42–3.72	2.72	1.44–5.16
**Health care profession**						
Yes	5 (37.70)	9 (51.30)	*Ref*			
No	235 (48.20)	68 (54.80)	1.67	0.55–5.06	1.02	0.24–4.30
**Chronic diseases**						
Yes	184 (48.70)	194 (51.30)	*Ref*			
No	56 (45.20)	68 (54.80)	0.86	(0.57–1.30)	0.93	0.57–1.52
**Attitude towards PPMs**						
Unfavourable	154 (69.70)	67 (30.30)	*Ref*			
Favourable	86 (30.60)	195 (69.40)	5.21	3.55–7.64	4.41	2.89–6.73
**Subjective Norm**						
Negative	128 (66.00)	66 (34.00)	*Ref*			
Positive	112 (36.40)	196 (63.60)	3.39	2.32–4.94	2.30	1.53–3.65
**Perceived Behavioural Control**						
Low	166 (55.00)	136 (45.00)	*Ref*			
High	74 (37.00)	126 (63.00)	2.07	1.44–2.99	1.72	1.13–2.61

### Factors associated with current practicing of PPMs

Results show that there were statistically significant differences in the current practicing of PPMs in relation to the level of education, having been a healthcare professional, Attitude towards PPMs, Subjective Norm, and Perceived Behavioural Control and the intention to practicing PPMs (*p* < 0.05).

Given these results, Table 5 shows unadjusted and adjusted logistic regression analyses of participants’ characteristics in relation to the current practicing of PPMs. In the adjusted model, only three of the four components of the TPB were statistically significant. Firstly, participants with a favourable Attitude were 62.10% less likely to report practicing PPMs (95% CI = 0.22–0.63). For Perceived Behavioural Control, participants who had reported a high Control were 40% less likely to practice PPMs (95% CI =0.36–0.98). As for the intention, participants who were intending to practice PPMs were 93.4% less likely to currently practice them (95% CI = 0.04–0.10).

**Table 5:** Unadjusted and adjusted binary logistic regression analyses of older adult participants predictors of the current practicing of personal preventive measures against the COVID-19 infection.

Predictors	Currently practicing PPMs		Unadjusted OR	95% CI	Adjusted OR	95% CI
	Not Practicing *N* (%) 261 (52.0)	Practicing *N* (%) 241 (48.0)				
**Age**						
< 70	157 (55.50)	126 (44.50)	*Ref*			
≥70 years	104 (47.50)	115 (52.50)	1.37	0.96–1.96	0.89	0.53–1.48
**Sex**						
Males	124 (50.40)	122 (49.60)	*Ref*			
Females	137 (53.50)	119 (46.50)	0.88	0.62–1.25	0.69	0.40–1.20
**Level of education**						
Read and write	62 (48.80)	83 (57.20)	*Ref*			
High school graduate	113 (50.70)	110 (49.30)	0.72	0.47–1.10	0.77	0.41–1.44
University/Postgraduate	86 (64.20)	48 (35.80)	0.41	0.25–1.04	0.49	0.23–1.05
**Health care profession**						
Yes	11 (78.60)	3 (21.40)	*Ref*			
No	250 (51.20)	238 (48.80)	0.28	0.07–1.04	0.17	0.02–1.14
**Chronic diseases**						
Yes	191 (50.50)	187 (49.50)	*Ref*			
No	70 (56.50)	54 (43.50)	0.78	0.52–1.18	1.20	0.66–2.18
**Attitude towards PPMs**						
Unfavourable	68 (30.80)	153 (69.20)	*Ref*			
Favourable	193 (68.70)	88 (331.30)	0.20	0.13–0.29	0.37	0.22–0.63
**Subjective Norm**						
Negative	73 (37.60)	121 (62.40)	*Ref*			
Positive	188 (61.00)	120 (36.00)	0.38	0.26–0.55	0.88	0.51–1.46
**Perceived Behavioural Control**						
Low	133 (44.00)	169 (56.00)	*Ref*			
High	128 (64.00)	72 (36.00)	0.44	0.30–0.63	0.60	0.36–0.98
**Intention to practice**						
Not intending	45 (18.80)	195 (81.20)	*Ref*			
Intending	216 (84.20)	46 (17.60)	0.04	0.03–0.07	0.06	0.04–0.10

## Discussion

Despite all scientific evidence, epidemiologically or biologically, on the positive effects of preventive measures against COVID-19, compliance with these measures remains variable. Cultural and behavioural factors play an important role in this variability. To the best of our knowledge, this is the first study to apply the TPB to predict adherence to public health preventive measures against the COVID-19 infection in the older adults’ vulnerable population. We examined both the intention as well as the actual current practicing of PPMs. It is also important to note that the collection of the data was past the date at which the government had scraped all restrictions, hence the results presented here show the attitudes and beliefs of the participants long after the extensive health campaigns and the COVID-19 public concern. Several important findings have been found regarding the intention and practice towards PPMs among the older adult Saudi population.

In our study, we found that over half the participants had intended to practice PPMs. These figures are very similar to those of studies in parts of Africa (22, 26), but are in stark contrast to those in the US where the national average intent to comply with four recommendations, namely, washing hands, social distancing, cough etiquette and stay at home was over 80% (27). These differences may be due to fatality differences between the two regions which may have played a role in the increased intention in the US.

### Factors associated with the intention to practice PPMs against COVID-19

Our study analysed factors predicting older adults’ intention to practice PPMs. We found that females were more likely to intend to practice PPMs, and those with higher education had an even greater odd of intention. This aligns with another that showed that women, and those with higher educational attainment, were more likely to adopt preventive measures (28). Rooted within the TPB, our findings also revealed that participants with a favourable Attitude were more likely to intend to practice PPMs. This underlines the impact of personal Attitudes on health behaviours, as confirmed by multiple studies, which found that positive Attitudes towards preventive measures significantly increased their adoption (29, 30). The role of social factors was also highlighted in our study. Participants with positive Subjective Norms were more likely to intend to practice PPMs against COVID-19, supporting the findings of a previous study which reported that positive social norms significantly influenced the uptake of preventive measures (31). Lastly, our study showed that older adults with high Perceived Behavioural Control were more likely to intend to practice PPMs against COVID-19, indicating the importance of individuals’ confidence in their ability to perform preventive behaviours. This complements the findings of a local study which found that perceived control significantly impacted the adoption of preventive measures (32). Overall, our findings confirm and expand upon existing literature, emphasizing the role of sex, education, Attitudes, social norms, and perceived control in influencing older adults’ intention to practice PPMs against COVID-19.

### Factors associated with the current practice of PPMs against COVID-19

With regards to the current practicing of PPMs, we found that, among the study participants, those with a favourable Attitude towards PPMs were about two-thirds less likely to practice PPMs. This contradicts the findings of previous studies, which demonstrated a positive correlation between Attitudes towards PPMs and their adoption (28, 31). We also found that participants who had a Perceived Behavioural Control were less likely to practice PPMs. This is in stark contrast to the findings of previous studies which highlighted that Perceived Behavioural Control was a significant predictive factor in the adoption of PPMs (30, 33). These studies suggested that individuals who believe they have the skills and resources to perform PPMs are more likely to do so. Interestingly, our study showed that participants who reported intending to practice PPMs were almost two times less likely to actually practice PPMs against COVID-19. This divergence between intention and behaviour contradicts the findings of a Turkish study which found a strong positive correlation between intentions and subsequent behaviour, particularly around health-related actions (34). It is interesting to find that in an Ethiopian study, although those who intended to practice PPMs were more likely to actually practice it, this association was not significant in their adjusted model (22). It is important though to note that the mean age in the first study was only 38.76 years, and in the second study it was 42.67 years, whereas in our study the mean age was 70.36 years. Notably, this divergence between intention and practice is not unheard of, the intention-behaviour gap is a phrase that describes the failure of intentions to be translated into actions (26). It maybe that the older adult population as a defined group possess specific behavioural characteristics unique to all other age groups. Overall, our unexpected findings highlight a complex interaction between Attitudes, Perceived Behavioural Control, intentions, and actual behaviour in the practice of PPMs among older adults. These findings suggest that other factors might be influencing the adoption of PPMs and that further research especially in the intention-practice gap is needed to fully understand these dynamics.

### Implications and future directions

Although literature focusing on the role of the TPB during the pandemic is flourishing, there is a lack of research focusing on the older adult population. The current finding in that intention does not necessarily mean practice is extremely important to investigate in further epidemiological studies for this particular vulnerable population. Public health agencies within the KSA should continue its efforts to raise awareness through communication plans regarding the importance of adopting these preventive measures even long after lifting all restrictions. These communication plans should incorporate the role of friends and family especially since social constructs have been found to be associated with adoption of preventive measures. Furthermore, epidemiologists should further conduct behavioural epidemiological studies that would further suggest practical recommendations for public health agencies and health authorities in the KSA. Funded national surveys managed by experienced epidemiologists would be particularly helpful in order to reach sample sizes of sufficient and generally representative quality. Also, given the current focus on the practice of Family and primary care physicians, particularly with the expansion in preventive clinics, the role of these healthcare practitioners is crucial in increasing both awareness and promoting the health of older adults and other vulnerable groups.

### Strengths and limitations

This is the first study to apply the TPB to the older adult population in the KSA within the context of preventive measures against COVID-19. However, the cross-sectional nature and the fact that both the intention and the actual practice were taken at the same time does not allow for any temporal associations to be made. Furthermore, the non-probability sampling technique, albeit consciously chosen due to the sensitive nature of our population, limits the generalisability of our results.

## Conclusion

This study has shown that long after lifting all restrictions against COVID-19 in the KSA, a little over half the older adult participants intend to practice PPMs. It also showed that females, participants with a higher education level, those with a favourable Attitude towards preventive measures, positive Subjective Norm and high Perceived Behavioural Control exhibited a higher odd of intending to practice PPMs. Most importantly, that intending to practice does not necessarily mean actual practicing. We recommend that public health agencies, health authorities and primary care physicians in the country to continue the efforts to raise awareness of proper preventive measures through customised communication plans. Also, the role of epidemiologists in terms of behavioural epidemiological studies should not be neglected.

## Data availability statement

The raw data supporting the conclusions of this article will be made available by the authors, without undue reservation.

## Ethics statement

The studies involving humans were approved by Institutional review board of Imam Abdulrahman Bin Faisal University (IRB-2022-01-294). The studies were conducted in accordance with the local legislation and institutional requirements. The participants provided their written informed consent to participate in this study.

## Author contributions

RA: Conceptualization, Data curation, Formal analysis, Methodology, Project administration, Software, Supervision, Validation, Visualization, Writing – original draft, Writing – review & editing. AmA: Conceptualization, Data curation, Project administration, Validation, Visualization, Writing – original draft, Writing – review & editing. LA: Conceptualization, Data curation, Methodology, Validation, Visualization, Writing – original draft, Writing – review & editing. SA: Conceptualization, Data curation, Methodology, Validation, Visualization, Writing – original draft, Writing – review & editing. ZA: Conceptualization, Data curation, Methodology, Validation, Visualization, Writing – original draft, Writing – review & editing. HA: Conceptualization, Data curation, Methodology, Validation, Visualization, Writing – original draft, Writing – review & editing. FA: Conceptualization, Data curation, Methodology, Validation, Visualization, Writing – original draft, Writing – review & editing. MA-S: Conceptualization, Data curation, Methodology, Validation, Visualization, Writing – original draft, Writing – review & editing. NoA: Conceptualization, Formal analysis, Methodology, Supervision, Validation, Visualization, Writing – original draft, Writing – review & editing. MS: Formal analysis, Investigation, Methodology, Project administration, Supervision, Writing – original draft, Writing – review & editing. AsA: Conceptualization, Formal analysis, Project administration, Supervision, Validation, Visualization, Writing – original draft, Writing – review & editing. NiA: Conceptualization, Formal analysis, Methodology, Supervision, Validation, Visualization, Writing – original draft, Writing – review & editing.
